# Eight-Channel AC Magnetosusceptometer of Magnetic Nanoparticles for High-Throughput and Ultra-High-Sensitivity Immunoassay

**DOI:** 10.3390/s18041043

**Published:** 2018-03-30

**Authors:** Jen-Jie Chieh, Wen-Chun Wei, Shu-Hsien Liao, Hsin-Hsein Chen, Yen-Fu Lee, Feng-Chun Lin, Ming-Hsien Chiang, Ming-Jang Chiu, Herng-Er Horng, Shieh-Yueh Yang

**Affiliations:** 1 Institute of Electro-Optical Science and Technology, National Taiwan Normal University, Taipei 116, Taiwan; clara6622@gmail.com (W.-C.C.); shliao@ntnu.edu.tw (S.-H.L.); willamsobain@gmail.com (M.-H.C.); 2 MagQu Co., Ltd., New Taipei 231, Taiwan; joseph.chen@magqu.com (H.-H.C.); yf.lee@magqu.com (Y.-F.L.); venus.lin@magqu.com (F.-C.L.); syyang@maqgu.com (S.-Y.Y.); 3 Departments of Neurology, National Taiwan University Hospital, Taipei 100, Taiwan; mjchiu@ntu.edu.tw; 4 Institute of Brain and Mind Sciences, College of Medicine, National Taiwan University, Taipei 100, Taiwan; 5 Department of Psychology, National Taiwan University, Taipei 106, Taiwan; 6 Graduate Institute of Biomedical Engineering and Bio-informatics, National Taiwan University, Taipei 106, Taiwan

**Keywords:** superconducting quantum interference device, magnetic nanoparticle, immunoassay

## Abstract

An alternating-current magnetosusceptometer of antibody-functionalized magnetic nanoparticles (MNPs) was developed for immunomagnetic reduction (IMR). A high-sensitivity, high-critical-temperature superconducting quantum interference device was used in the magnetosusceptometer. Minute levels of biomarkers of early-stage neurodegeneration diseases were detectable in serum, but measuring each biomarker required approximately 4 h. Hence, an eight-channel platform was developed in this study to fit minimal screening requirements for Alzheimer’s disease. Two consistent results were measured for three biomarkers, namely Aβ40, Aβ42, and tau protein, per human specimen. This paper presents the instrument configuration as well as critical characteristics, such as the low noise level variations among channels, a high signal-to-noise ratio, and the coefficient of variation for the biomarkers’ IMR values. The instrument’s ultrahigh sensitivity levels for the three biomarkers and the substantially shorter total measurement time in comparison with the previous single- and four-channels platforms were also demonstrated in this study. Thus, the eight-channel instrument may serve as a powerful tool for clinical high-throughput screening of Alzheimer’s disease.

## 1. Introduction

To accurately screen for and diagnose diseases at early stages, precise immunoassays such as a single-molecule array [1], mesoscale discovery assay [2], and single-molecule count [3] have been reported to exhibit higher sensitivity than current clinical methods such as enzyme-linked immunosorbent assays (ELISAs) and radioimmunoassay. However, limitations have also been found for precise immunoassays with regard to operational complexity and interference. Immunomagnetic reduction (IMR), entailing the use of a magnetic reagent comprising a solvent and bioprobe-coated magnetic nanoparticles (MNPs) as labeling markers, was recently proposed to address the mentioned limitations; the advantages of IMR include a wash-free assay, high specificity, one-antibody utility, long lifetime, and biosafety [4]. Preparation for IMR involves mixing a magnetic reagent and liquid sample with target biomolecules. Subsequently, an IMR instrument is used to measure the variation of the alternating-current (ac) magnetic susceptibility *χ*_ac_ of the mixture under a bioconjugation process. During bioconjugation, target molecules connect to bioprobes on MNPs, and a large magnetic cluster forms. With regard to the ac excitation field (i.e., *χ*_ac_), the magnetic cluster exhibits a substantially lower response than a single MNP. An intuitive explanation of this phenomenon is that the rotational velocity, the direction of which is influenced by the ac magnetic field, is slower for a large magnetic cluster than for a single, smaller MNP. Thus, the formation of a large magnetic cluster results in the reduction of *χ*_ac_. Further reduction of *χ*_ac_ occurs as the concentration of target molecules increases. In addition, the bounded nontarget molecules, so-called interference materials, may spin away from the MNPs because the centrifugal force generated by the rotation of MNPs is larger than the weak binding force of a nonspecific bioconjugation process. In a previous study, an excitation frequency of approximately 20 kHz was chosen because at this frequency, the magnetic moments of single MNPs with hydrodynamic diameters of approximately 50 nm could optimally oscillate with the external ac magnetic field, and the magnetic moments of the particle clusters were almost immobile [5]. These phenomena contribute to the high specificity of IMR compared with other MNP immunoassay methods such as the relaxation time method based on the same types of bioprobe-coated MNPs [6]. 

In IMR, first-order pick-up coils may be utilized to measure the variation of *χ*_ac_ for a mixture, but signal processing is required to determine the minimum detectable concentration and, thus, immunoassay sensitivity. Electronic-type IMR instruments, comprising electronic amplifiers and filters, usually achieve immunoassay sensitivity at the sub-parts-per-billion level [7,8]. However, this level of immunoassay sensitivity is only slightly better or similar to that achievable with ELISA and other precision immunoassay methods. Because superconducting quantum interference devices (SQUIDs) are highly sensitive magnetic sensors, most magnetic immunoassay methods [9,10,11,12,13,14,15] utilize SQUID sensors. SQUID-type IMR instruments have unique measurement configurations and minor differences in the mechanism of switching from one channel [11] to four channels [12]; thus, such instruments have enhanced immunoassay sensitivity of up to 10^−2^ parts per trillion. At this level of sensitivity, Alzheimer’s disease (AD)-related biomarkers are detectable in the plasma of patients. For patients with neurodegenerative diseases, including AD, only minute biomarker amounts are present in plasma because of the barrier between the central nervous system and blood system. Hence, IMR is the only available clinical method of plasma diagnosis [11,12,13,14,15]. AD is prevalent in many developed countries and creates heavy financial and care burdens for many families and societies because no drug for recovery exists. The detection of AD biomarkers before early-stage AD and mild cognitive impairment (MCI) may enable early treatment with currently available drugs and thus prevention of disease progression. 

For a SQUID-type IMR instrument with multiple channels, the switching mechanism could theoretically divide the measurement time of one biomarker into that of each channel in several switch cycles. In this study, sufficient switch cycles were applied to classify the measured data points of all switch cycles according to the initial and final states of the bioconjugation process. Hence, several biomarkers could be screened simultaneously in different channels, and the cycle measurement time of each channel dominated the total time. The measurement time of each channel relied on real measurement time and automatically adjusted time for the lock and unlock states of the SQUID sensor. The switching mechanism during switching introduced the large noise to the SQUID sensor as the unlock states, and then prolonged the automatically adjusted time, processed by the software program. For example of the four-channel platform [12], this switching mechanism always introduced too large noise level because the connection wire of the input coil surrounding the SQUID sensor became unconnected like the antenna during the short switch time, and then the SQUID sensor was interfered by the environmental noise to be the unlock state. Hence, the switching mechanism described in this study was developed to enhance the SQUID-type IMR instrument with eight channels. 

## 2. Experiments

**Figure 1:**
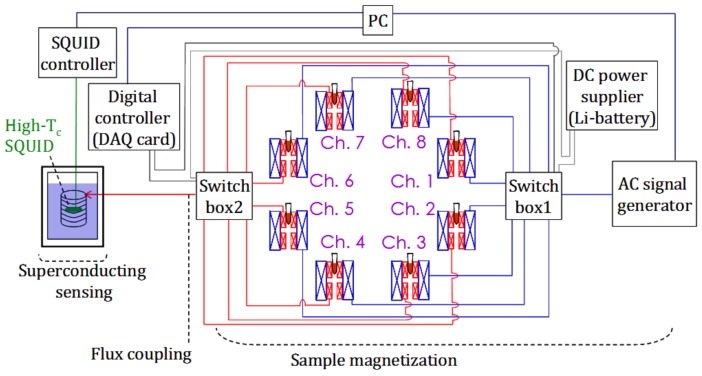
Configuration of the eight-channel ac magnetosusceptometer for MNPs.

The eight-channel ac magnetosusceptmeter for MNPs developed in this study comprised three components: sample magnetization, flux coupling, and superconducting sensing (Figure 1). 

**Figure 2:**
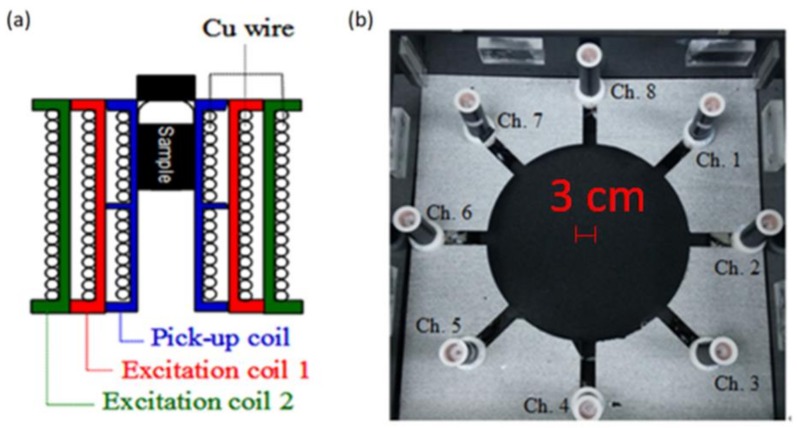
The eight channels of pick-up coils: (**a**) Assembly of one set of coils and (**b**) array of eight coil sets.

The sample magnetization system consisted of eight sets of coils. Each set of coils comprised two excitation coils (referred to as excitation coils 1 and 2) and one pick-up coil. These three coils were assembled coaxially, with the pick-up coil being the innermost coil (Figure 2a). The pick-up coil was an axial gradiometer. The sample that was mixed with the reagent was located in the upper section of the pick-up coil. The eight sets of coils were arrayed as a right octagon to ensure that all sets of coils were geometrically identical. To prevent electromagnetic cross-talk among coil sets, neighboring coil sets were positioned 15 cm apart (Figure 2b).

For each coil set, excitation coils were driven using an ac signal generator, which generated two independent ac voltages at two frequencies, f_1_ and f_2_, to excitation coils 1 and 2, respectively. The mixed frequency of f_1_ + 2f_2_ at approximately 20 kHz was used to suppress signals associated with no samples [5]. The ac magnetic signal that resulted from magnetized samples was detected using the pick-up coil and guided to the flux-coupling component. The eight coil sets were activated sequentially. To manipulate the activation of each coil set, electric switches were cascaded between the ac signal generator and excitation coils as well as between the pick-up coil and flux-coupling component, as indicated by switch boxes 1 and 2 (Figure 1).

**Figure 3:**
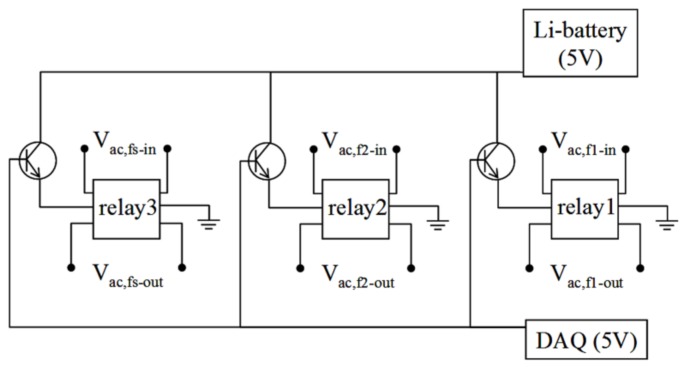
Circuits for electric switches used in the eight-channel AC magnetosusceptometer for MNPs.

The circuits of the electric switches are illustrated in Figure 3. Each coil set contained three switches, and each switch comprised one low-noise bipolar junction transistor (BJT) (C1815, UTC, New Taipei City, Taiwan) and one low-noise relay (G6K-2P-Y, Omron Corporation, Kyoto, Japan). The low noise BJT was as a buffer to isolate the DAQ noise from pickup coil and the input coil surrounding the SQUID sensor. It efficiently improved the automatically adjusted time for the unlock states of the SQUID sensor. Relays 1 and 2 (Figure 3) transferred ac voltages from the ac signal generator to excitation coils 1 and 2, respectively. Relay 3 transferred the ac signal from the sample to the flux-coupling component. Activation of the relay was controlled with the BJT, which had a power of 5 V and contained a data-acquisition (DAQ) card (PCI-6221, National Instruments, Austin, TX, USA). The DAQ card output a direct current (dc) of 5 V to the BJT, and the dc voltage generated by the Li battery was applied to the relay. The relay was activated, and either the ac voltage from the ac signal generator was transferred to the excitation coil, or the sample-induced ac signal was transferred to the flux-coupling component. Once the 5-V dc from the DAQ card was stopped, the relay was also stopped. Subsequently, no ac voltage or signal was transferred through the relay. Relays 1 and 2 were inside switch box 1, and relay 3 was inside switch box 2.

The flux-coupling component comprised a pair of twisted wires. One end of the wires was connected to the output ports of relay 3 (i.e., Vac,fs-out in Figure 3). The other end of the wires was linked to a coil. Once Vac,fs-out was activated, an ac electric current I was induced along the wires, and an ac magnetic field B was generated at the coil terminal. The ac magnetic field B at the coil terminal was detected using a high-critical-temperature (high-T_c_) SQUID magnetometer (v 5.0, JSQ GmbH), which was part of the superconducting sensing component.

The SQUID magnetometer and coil terminal were immersed in liquid nitrogen. The dewar was posited inside an electromagnetically shielded box, which exhibited a shielding factor of 100 dB at an operating frequency of approximately 20 kHz. The SQUID magnetometer was controlled with a controller (v 5.0, JSQ GmbH), the output signals of which were guided to a personal computer. 

Three reagent types were used. The reagents, comprising MNPs with various bioprobes, bound specifically with β-amyloid 40 (Aβ40) (MF-AB0-0060, MagQu, Ltd., New Taipei City, Taiwan), β-amyloid 42 (Aβ42) (MF-AB2-0060, MagQu Ltd.), and tau protein (MF-TAU-0060, MagQu Ltd.). The utilized MNPs with approximately 50 nm in hydrodynamic diameter were composed of a Fe_3_O_4_ core and dextran in its shell [16]. For Aβ40 and a tau protein assay, an 80-μL reagent was mixed with a 40-μL sample. A 60-μL reagent was mixed with a 60-μL sample for assaying Aβ42. After the reagent was mixed with the sample, the real-time ac magnetic signal (i.e., IMR signal) was recorded to determine the reduction percentage. For each sample, IMR signal detection procedures were performed in duplicate.

## 3. Results and Discussion

To evaluate the specification differences among the channels, both nonsamples and a biomarker-free mixture without bioconjuation-induced variation of *χ*_ac_ were separately measured in all eight channels. For these measurements, the biomarker-free mixture comprised an 80-μL Aβ40 reagent and a 40-μL phosphate buffered saline solution. 

**Figure 4:**
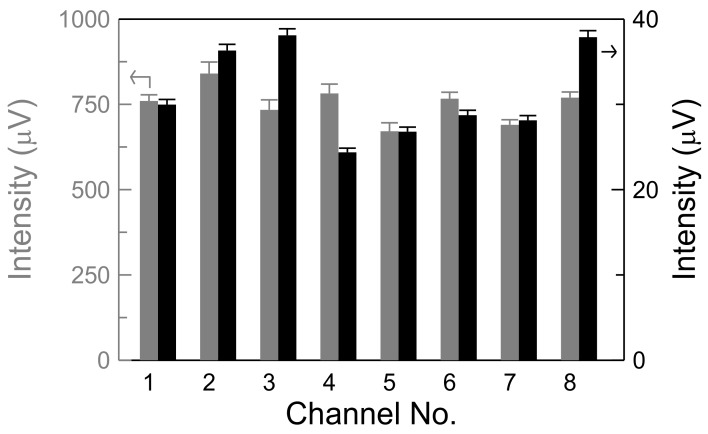
The consistency study of channels with the measurement of noise levels (black bars) and biomarker-free mixture (gray bars) without the bioconjugation process.

The recording time was approximately 1 min for each channel, much longer than the total measurement time of tens of seconds. Figure 4 shows the recorded signal intensity of the biomarker-free mixture, represented by the gray bar, and the noise level, represented by the black bar. The observation frequency at 20 kHz was within the approximately 1-MHz bandwidth of the controller. Four critical findings validated consistency among these eight channels. First, the time-independent intensity, representative of *χ*_ac_ before or without the bioconjugation of target molecules (denoted as *χ*_ac__,0_), exhibited a variance of only 7.1%. Second, the noise level ranged from 20 to 40 μV, similar to the noise level for the shorter measurement time of tens of seconds. Third, similar standard deviations were identified between the output signals and the noise level. Fourth, the signal-to-noise ratio for all channels was approximately 24.5, which is sufficiently high for reliable measurement. Notably, all reagents exhibited the same results for the biomarker-free mixture (Figure 4) because the only differences among the three reagents were the bioprobes, which were coated on the same batch of MNPs.

Subsequently, the assay performance of each channel was evaluated for the three biomarkers Aβ40, Aβ42, and tau protein. These biomarkers are related to AD and other neurodegenerative diseases. To achieve a high-throughput assay, the measurement guidelines were set that three biomarkers per person specimen should be measured for two consistent duplicates. Hence, each biomarker was mainly measured with two channels and supplementally with one channel, except for the tau protein—the most indicative biomarker of neurodegenerative diseases such as AD—for which two auxiliary channels were used. Channels 1 and 2 were dedicated to the tau protein; channels 3 and 4 were used for Aβ42 primarily and the tau protein secondarily; channels 7 and 8 were dedicated to Aβ40; channel 5 was reserved for the reference sample for calibration (Figure 4); and channel 6 was used as a support for the measurement of Aβ40 or Aβ42. 

Immunoassay performance was evaluated using the coefficient of variation (CV) and the relations between known concentrations of target molecules and IMR values. The IMR value can be defined as follows [12,13,14,15]:

(1) $$IMR(\%)=\frac{\chi_{\mathrm{ac},0}-\chi_{\mathrm{ac},\varphi }}{\chi_{\mathrm{ac},0}}\times 100\%$$

where *χ*_ac__,o_ and *χ*_ac__,_*_Φ_* are the *χ*_ac_ of the mixture before and after the bioconjugation of target molecules, respectively, the concentration of which is denoted as *φ*. 

**Figure 5:**
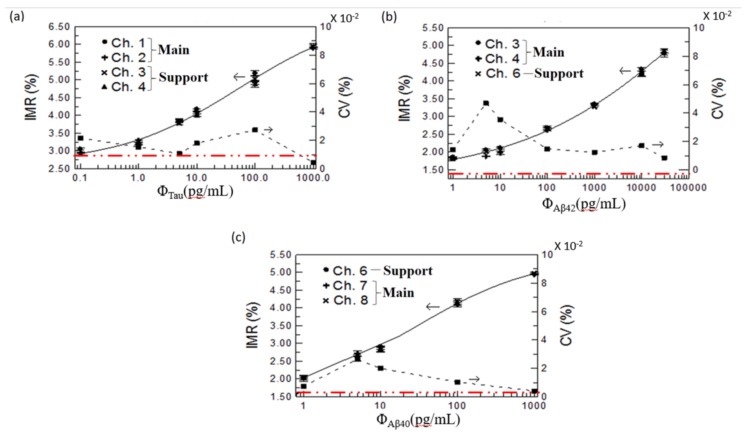
Relationship between the concentrations of (**a**) Aβ40; (**b**) Aβ42; and (**c**) tau protein and the measurement parameters of IMR and CV values in and among channels. IMR with a low detection limit for the various biomarkers is marked with red doted lines.

For three measurement rounds of the same biomarker concentration obtained from each main or support channel, the average and standard deviations of IMR values are plotted on the left axes in Figure 5a–c for the tau protein, Aβ42, and Aβ40, respectively. The CV, defined as the ratio of the standard deviation to the average value, was used as the stability indicator in comparison with all obtained IMR values from the various channels for each biomarker. The CV values are plotted on the right axes in Figure 5a–c for the tau protein, Aβ42, and Aβ40, respectively. All CV values for the biomarkers in concentration ranges lower than 5% validated high consistency for the IMR values obtained from the main and support channels. The biomarker ranges for the tau protein, Aβ42, and Aβ40 were 1–30,000, 0.1–1000 pg/mL, and 1–1000 pg/mL, respectively. 

To analyze the biomarker concentration in human plasma by using the obtained IMR data from all main or support channels, the experimental relations between the average IMR values obtained from all main or support channels and the known biomarker concentrations were fitted as standard curves, marked as solid lines in Figure 5a–c. The standard curves of all biomarkers followed the same logistic function [12,13,14,15]:

(2) $$IMR(\%)=\frac{A-B}{1+{\left(\frac{\varphi }{\varphi_{o}}\right)}^{\gamma }}+B$$

where *A*, *B*, *φ_o_*, and *γ* denote fitting parameters and *φ* denotes the biomarker concentration. All fitted values of *A*, *B*, *φ_o_*, and *γ* as well as the coefficient of determination R^2^ for the biomarkers Aβ40, Aβ42, and tau protein are listed in Table 1.

The value of *A* in Equation (2) represents the IMR signals without biomarkers. *A* is the lowest level for the IMR signals. Once the biomarker concentration becomes infinite, the IMR signals approach B. Thus, *B* is the saturated IMR signal value at high biomarker concentrations. *φ_o_* corresponds to the biomarker concentration at which the IMR signals are (*A* + *B*)/2. The power *γ* is the slope d[IMR(%)]/d*φ* where *φ* is at *φ_o_*. 

The critical metric of immunoassay performance, the concentration at a low detection limit, was derived from IMR of a low detection limit using Equation (2). In this study, IMR of a low detection limit for one biomarker in spite of the measurement channel was defined as A + 3 × CV_max_, and plotted with the red dotted line in Figure 5. Here, CV_max_ is the maximum CV value in the experimental range of test concentration, part of detectable concentrations. For the detection on the low concentration, the IMR value was close to the minimum IMR of no biomarkers, i.e., A value, and the definition based on three times of the IMR variation among main or support channels was strict to discrimination from the worst case of zero concentration. In addition, to validate the reliability of a low detection limit, the average intensity at a low detection limit should be larger than the noise level for each main or support channel for each biomarker. The average intensity of a low detection limit was *χ*_ac,*Φ*_, and could be obtained from *χ*_ac,0_, based on the biomarker-free mixture in Figure 4, by using Equation (1). 

For the detection of Aβ40, first, IMR of a low detection limit was obtained as (1.51 + 3 × 0.025)% = 1.585% due to 0.025% of the maximum CV value occurring at 5.5 pg/mL in Figure 5c and 1.51% of the parameter A in Table 1. Second, the concentration of a low detection limit was determined to be approximately 0.03 pg/mL. Third, the average intensities at a low detection limit, relative to *χ*_ac__,_*_Φ_*, were 770.5 × (100% − 1.585%) = 758.3 µV, 692.6 × (100% − 1.585%) = 681.6 µV, and 771.0 × (100% − 1.585%) = 758.8 µV for channels 6, 7, and 8, respectively. These values were clearly higher than the noise level plotted relative to the right axis in Figure 4. Similarly, the IMR values and concentration of low detection limits in assaying Aβ42 and tau protein were determined to be 1.39% and 2.90% and 3 × 10^–4^ pg/mL and 0.06 pg/mL, respectively. The IMR values of low detection-limits for the biomarkers are marked with the red doted lines in Figure 5a–c. The concentrations in the plasma of healthy people and patients with early-stage AD were found to be, respectively, (71.4 ± 30.8) pg/mL and (38.2 ± 9.2) pg/mL for the Aβ40 biomarker, (15.3 ± 1.6) pg/mL and (18.6 ± 1.5) pg/mL for the Aβ42 biomarker, and (16.2 ± 9.1) pg/mL and (53.6 ± 22.8) pg/mL for the tau protein biomarker [13,14,15]. Hence, the concentrations of the low detection limits were much lower than the concentrations found in healthy and early-stage AD patients. Therefore, the eight-channel ac magnetosusceptometer is sufficiently sensitive to detect and quantify these biomarkers in human plasma in screenings for early-stage AD or MCI.

The total measurement time was also a critical factor for evaluation. For each of the three biomarkers at a concentration of several picograms per milliliter, the measurement times were up to approximately 4 h when the single-channel platform was used, and they were theoretically only dependent on the conjugation process. Hence, when the multichannel platform with the switching mechanism was used, the measurement time of one biomarker, (i.e., one round) was still approximately 4 h. The total measurement time should be defined as that for the total specimens and should be compared with the ratio of the total specimens over the utilized channel number. For example, the total measurement time of seven specimens was approximately 4 h over one round for the eight-channel platform, with one channel used for calibration; 12 h over three rounds for the previously developed four-channel platform, with one channel used for calibration; and 28 h over seven rounds for the previously developed single-channel platform involving a complicated calibration process. 

## 4. Conclusions

An eight-channel ac magnetosusceptometer for MNPs was developed for high-throughput screening. The developed magnetosusceptometer effectively measured seven biomarkers per round at a high sensitivity, detecting quantities much lower than 1 pg/mL. The instrument design involved a switching mechanism and high-T_c_ SQUID. High consistency was demonstrated among channels, with only 7.1% variation for the biomarker-free mixture. The developed magnetosusceptometer exhibited a signal-to-noise ratio of approximately 24.5. The instrument proved effective for the detection of minute biomarker amounts in serum, particularly for three biomarkers of AD used in this study. Results were verified in duplications of measured data. 

## Figures and Tables

**Table 1:** Fitted values of parameters *A*, *B*, *φ_o_*, and *γ* from Equation (2), and coefficient of determination R^2^ for the relationships between IMR signals and biomarker concentrations.

	Parameter	*A*	*B*	*φ_o_*	*γ*	R^2^
Biomarker						
Aβ40		1.51	5.46	29.45	0.55	0.998
Aβ42		1.29	10.42	228561.4	0.23	0.999
Tau protein		2.64	6.89	53.09	0.42	0.998
